# Supplementary material for: Effects of ground robot manipulation on hen floor egg reduction, production performance, stress response, bone quality, and behavior
Source: PLoS One. 2022 Apr 22;17(4):e0267568. doi: 10.1371/journal.pone.0267568 (PMC9032375; doi:10.1371/journal.pone.0267568)
Supplement: S1 Dataset — (DOCX) [file pone.0267568.s001.docx]

**Minimal Data Set-Figures**

| Fig. # | Mean | S.D. | S.E. | Statistical methods used | P value | # samples |
| --- | --- | --- | --- | --- | --- | --- |
| **Fig 3** |  |  |  |  |  |  |
| Egg mass (g/egg) | | | | Two-way ANOVA using PROC MIXED statement, LSD comparison |  |  |
| Nest egg | 62.7 | 1.5 | 0.3 |  | 0.05 | 24 |
| Floor egg | 61.5 | 2.3 | 0.5 |  |  | 24 |
| Overall | 62.5 | 1.3 | 0.3 |  |  | 24 |
| **Fig 4a** |  |  |  |  |  |  |
| Live body weight (kg/bird) | | | | One-way ANOVA using PROC MIXED statement, LSD comparison |  |  |
| w/o robot running | 1.83 | 0.06 | 0.03 |  | 0.38 | 4 |
| w/ one-week robot running | 1.84 | 0.06 | 0.03 |  |  | 4 |
| w/ two-week robot running | 1.83 | 0.06 | 0.03 |  |  | 4 |
| **Fig 4b** |  |  |  |  |  |  |
| Litter moisture content (%) | | | |  |  |  |
| w/o robot running | 18.2 | 8.9 | 3.6 |  | 0.41 | 6 |
| w/ one-week robot running | 16.4 | 6.4 | 2.6 |  |  | 6 |
| w/ two-week robot running | 14.5 | 1.2 | 0.5 |  |  | 6 |
| **Fig 5a** |  |  |  |  |  |  |
| Frequency of number of birds simultaneously using a nest box during 7:00 to 13:00 in weeks 35-38 (%)-w/o robot running | | | | $-$ |  |  |
| 1 | 80.3 | 5.9 | 0.8 |  | $-$ | 52 |
| 2 | 11.0 | 4.0 | 0.5 |  |  | 52 |
| 3 | 4.2 | 2.0 | 0.3 |  |  | 52 |
| 4 | 3.8 | 1.9 | 0.3 |  |  | 52 |
| ≥5 | 0.8 | 0.8 | 0.1 |  |  | 52 |
| Frequency of number of birds simultaneously using a nest box during 7:00 to 13:00 in weeks 35-38 (%)-w/ one-week robot running | | | | $-$ |  |  |
| 1 | 82.3 | 6.0 | 0.8 |  | $-$ | 52 |
| 2 | 10.7 | 4.0 | 0.6 |  |  | 52 |
| 3 | 3.3 | 1.5 | 0.2 |  |  | 52 |
| 4 | 3.1 | 1.7 | 0.2 |  |  | 52 |
| ≥5 | 0.5 | 0.6 | 0.1 |  |  | 52 |

| Fig. # | Mean | S.D. | S.E. | Statistical methods used | P value | # samples |
| --- | --- | --- | --- | --- | --- | --- |
| **Fig 5a** |  |  |  |  |  |  |
| Frequency of number of birds simultaneously using a nest box during 7:00 to 13:00 in weeks 35-38 (%)-w/ two-week robot running | | | | $-$ |  |  |
| 1 | 79.0 | 6.1 | 0.9 |  | $-$ | 52 |
| 2 | 11.4 | 4.2 | 0.6 |  |  | 52 |
| 3 | 4.6 | 2.0 | 0.3 |  |  | 52 |
| 4 | 4.3 | 1.8 | 0.2 |  |  | 52 |
| ≥5 | 0.8 | 0.7 | 0.1 |  |  | 52 |
| **Fig 5b** |  |  |  |  |  |  |
| Frequency of number of birds simultaneously using a nest box during 7:00 to 13:00 in weeks 40-43 (%)-w/o robot running | | | | $-$ |  |  |
| 1 | 80.0 | 4.5 | 0.6 |  | $-$ | 66 |
| 2 | 13.2 | 4.0 | 0.5 |  |  | 66 |
| 3 | 3.2 | 1.2 | 0.1 |  |  | 66 |
| 4 | 3.1 | 1.3 | 0.2 |  |  | 66 |
| ≥5 | 0.4 | 0.3 | 0.1 |  |  | 66 |
| Frequency of number of birds simultaneously using a nest box during 7:00 to 13:00 in weeks 40-43 (%)-w/ one-week robot running | | | | $-$ |  |  |
| 1 | 83.2 | 4.7 | 0.6 |  | $-$ | 66 |
| 2 | 10.3 | 3.2 | 0.4 |  |  | 66 |
| 3 | 3.2 | 1.3 | 0.2 |  |  | 66 |
| 4 | 3.0 | 1.3 | 0.2 |  |  | 66 |
| ≥5 | 0.4 | 0.3 | 0.1 |  |  | 66 |
| Frequency of number of birds simultaneously using a nest box during 7:00 to 13:00 in weeks 40-43 (%)-w/ two-week robot running | | | | $-$ |  |  |
| 1 | 81.7 | 6.1 | 0.7 |  | $-$ | 66 |
| 2 | 12.0 | 4.4 | 0.5 |  |  | 66 |
| 3 | 3.1 | 1.6 | 0.2 |  |  | 66 |
| 4 | 2.8 | 1.6 | 0.2 |  |  | 66 |
| ≥5 | 0.4 | 0.4 | 0.1 |  |  | 66 |
| **Fig 5c** |  |  |  |  |  |  |
| Frequency of number of birds simultaneously using a nest box in a day in weeks 35-38 (%)-w/o robot running | | | | $-$ |  |  |
| 1 | 81.0 | 5.8 | 0.8 |  | $-$ | 52 |
| 2 | 10.2 | 3.5 | 0.5 |  |  | 52 |
| 3 | 4.1 | 2.0 | 0.3 |  |  | 52 |
| 4 | 3.8 | 2.0 | 0.3 |  |  | 52 |
| ≥5 | 0.8 | 0.8 | 0.1 |  |  | 52 |

| Fig. # | Mean | S.D. | S.E. | Statistical methods used | P value | # samples |
| --- | --- | --- | --- | --- | --- | --- |
| **Fig 5c** |  |  |  |  |  |  |
| Frequency of number of birds simultaneously using a nest box in a day in weeks 35-38 (%)-w/ one-week robot running | | | | $-$ |  |  |
| 1 | 80.4 | 7.0 | 1.0 |  | $-$ | 52 |
| 2 | 11.1 | 4.4 | 0.6 |  |  | 52 |
| 3 | 3.9 | 2.0 | 0.3 |  |  | 52 |
| 4 | 3.8 | 2.2 | 0.3 |  |  | 52 |
| ≥5 | 0.9 | 1.0 | 0.1 |  |  | 52 |
| Frequency of number of birds simultaneously using a nest box in a day in weeks 35-38 (%)-w/ two-week robot running | | | | $-$ |  |  |
| 1 | 78.4 | 6.6 | 0.9 |  | $-$ | 52 |
| 2 | 11.2 | 4.4 | 0.6 |  |  | 52 |
| 3 | 4.6 | 1.8 | 0.2 |  |  | 52 |
| 4 | 4.4 | 1.7 | 0.2 |  |  | 52 |
| ≥5 | 1.1 | 1.3 | 0.2 |  |  | 52 |
| **Fig 5d** |  |  |  |  |  |  |
| Frequency of number of birds simultaneously using a nest box in a day in weeks 40-43 (%)-w/o robot running | | | | $-$ |  |  |
| 1 | 82.5 | 4.0 | 0.5 |  | $-$ | 66 |
| 2 | 11.2 | 3.2 | 0.4 |  |  | 66 |
| 3 | 3.0 | 1.1 | 0.1 |  |  | 66 |
| 4 | 2.8 | 1.1 | 0.1 |  |  | 66 |
| ≥5 | 0.4 | 0.3 | 0.1 |  |  | 66 |
| Frequency of number of birds simultaneously using a nest box in a day in weeks 40-43 (%)-w/ one-week robot running | | | | $-$ |  |  |
| 1 | 82.6 | 4.9 | 0.6 |  | $-$ | 66 |
| 2 | 11.2 | 3.5 | 0.4 |  |  | 66 |
| 3 | 2.9 | 0.9 | 0.1 |  |  | 66 |
| 4 | 2.8 | 1.0 | 0.1 |  |  | 66 |
| ≥5 | 0.3 | 0.3 | 0.1 |  |  | 66 |
| Frequency of number of birds simultaneously using a nest box in a day in weeks 40-43 (%)-w/ two-week robot running | | | | $-$ |  |  |
| 1 | 81.2 | 4.8 | 0.6 |  | $-$ | 66 |
| 2 | 11.1 | 3.3 | 0.4 |  |  | 66 |
| 3 | 3.6 | 1.5 | 0.2 |  |  | 66 |
| 4 | 3.4 | 1.7 | 0.2 |  |  | 66 |
| ≥5 | 0.7 | 0.9 | 0.1 |  |  | 66 |

| Fig. # | Mean | S.D. | S.E. | Statistical methods used | P value | # samples |
| --- | --- | --- | --- | --- | --- | --- |
| **Figs 6a and 6b** |  |  |  |  |  |  |
| Hourly time spent in nest boxes from 6:00 to 21:00 in weeks 35-38 (min/bird/h)-w/o robot running | | | | $-$ |  |  |
| 6 | 0.57 | 0.42 | 0.06 |  | $-$ | 52 |
| 7 | 1.04 | 0.36 | 0.05 |  |  | 52 |
| 8 | 1.33 | 0.65 | 0.09 |  |  | 52 |
| 9 | 1.27 | 0.69 | 0.10 |  |  | 52 |
| 10 | 1.28 | 0.70 | 0.10 |  |  | 52 |
| 11 | 1.20 | 0.79 | 0.11 |  |  | 52 |
| 12 | 1.06 | 0.92 | 0.13 |  |  | 52 |
| 13 | 0.82 | 0.83 | 0.11 |  |  | 52 |
| 14 | 0.79 | 0.83 | 0.12 |  |  | 52 |
| 15 | 0.79 | 0.92 | 0.13 |  |  | 52 |
| 16 | 0.70 | 0.72 | 0.10 |  |  | 52 |
| 17 | 0.63 | 0.72 | 0.10 |  |  | 52 |
| 18 | 0.46 | 0.50 | 0.07 |  |  | 52 |
| 19 | 0.55 | 0.60 | 0.08 |  |  | 52 |
| 20 | 0.60 | 0.75 | 0.10 |  |  | 52 |
| 21 | 11.55 | 12.76 | 1.77 |  |  | 52 |
| Hourly time spent in nest boxes from 6:00 to 21:00 in weeks 35-38 (min/bird/h)-w/ one-week robot running | | | | $-$ |  |  |
| 6 | 0.71 | 0.66 | 0.09 |  | $-$ | 52 |
| 7 | 1.17 | 0.62 | 0.09 |  |  | 52 |
| 8 | 1.11 | 0.57 | 0.08 |  |  | 52 |
| 9 | 1.04 | 0.50 | 0.07 |  |  | 52 |
| 10 | 1.11 | 0.45 | 0.06 |  |  | 52 |
| 11 | 1.03 | 0.55 | 0.08 |  |  | 52 |
| 12 | 0.81 | 0.55 | 0.08 |  |  | 52 |
| 13 | 0.83 | 0.54 | 0.07 |  |  | 52 |
| 14 | 0.83 | 0.55 | 0.08 |  |  | 52 |
| 15 | 0.73 | 0.56 | 0.08 |  |  | 52 |
| 16 | 0.76 | 0.58 | 0.08 |  |  | 52 |
| 17 | 0.58 | 0.53 | 0.07 |  |  | 52 |
| 18 | 0.49 | 0.43 | 0.06 |  |  | 52 |
| 19 | 0.50 | 0.52 | 0.07 |  |  | 52 |
| 20 | 0.93 | 1.68 | 0.23 |  |  | 52 |
| 21 | 11.96 | 12.55 | 1.74 |  |  | 52 |

| Fig. # | Mean | S.D. | S.E. | Statistical methods used | P value | # samples |
| --- | --- | --- | --- | --- | --- | --- |
| **Figs 6a and 6b** |  |  |  |  |  |  |
| Hourly time spent in nest boxes from 6:00 to 21:00 in weeks 35-38 (min/bird/h)-w/ two-week robot running | | | | $-$ |  |  |
| 6 | 1.04 | 0.75 | 0.10 |  | $-$ | 52 |
| 7 | 1.48 | 0.65 | 0.09 |  |  | 52 |
| 8 | 1.57 | 0.73 | 0.10 |  |  | 52 |
| 9 | 1.38 | 0.64 | 0.09 |  |  | 52 |
| 10 | 1.33 | 0.57 | 0.08 |  |  | 52 |
| 11 | 1.27 | 0.79 | 0.11 |  |  | 52 |
| 12 | 1.11 | 0.62 | 0.09 |  |  | 52 |
| 13 | 1.20 | 0.69 | 0.10 |  |  | 52 |
| 14 | 1.46 | 0.93 | 0.13 |  |  | 52 |
| 15 | 1.22 | 0.86 | 0.12 |  |  | 52 |
| 16 | 1.36 | 1.17 | 0.16 |  |  | 52 |
| 17 | 1.09 | 0.87 | 0.12 |  |  | 52 |
| 18 | 0.96 | 0.82 | 0.11 |  |  | 52 |
| 19 | 0.80 | 0.61 | 0.09 |  |  | 52 |
| 20 | 0.84 | 0.82 | 0.11 |  |  | 52 |
| 21 | 14.78 | 15.84 | 2.20 |  |  | 52 |
| **Figs 6c and 6d** |  |  |  |  |  |  |
| Hourly time spent in nest boxes from 6:00 to 21:00 in weeks 40-43 (min/bird/h)-w/o robot running | | | | $-$ |  |  |
| 6 | 0.79 | 0.61 | 0.07 |  | $-$ | 66 |
| 7 | 1.24 | 0.70 | 0.09 |  |  | 66 |
| 8 | 1.30 | 0.51 | 0.06 |  |  | 66 |
| 9 | 1.09 | 0.42 | 0.05 |  |  | 66 |
| 10 | 1.07 | 0.57 | 0.07 |  |  | 66 |
| 11 | 0.69 | 0.54 | 0.07 |  |  | 66 |
| 12 | 0.58 | 0.47 | 0.06 |  |  | 66 |
| 13 | 0.51 | 0.42 | 0.05 |  |  | 66 |
| 14 | 0.51 | 0.39 | 0.05 |  |  | 66 |
| 15 | 0.48 | 0.48 | 0.06 |  |  | 66 |
| 16 | 0.44 | 0.43 | 0.05 |  |  | 66 |
| 17 | 0.42 | 0.35 | 0.04 |  |  | 66 |
| 18 | 0.48 | 0.31 | 0.04 |  |  | 66 |
| 19 | 0.50 | 0.39 | 0.05 |  |  | 66 |
| 20 | 0.56 | 0.58 | 0.07 |  |  | 66 |
| 21 | 8.59 | 9.60 | 1.18 |  |  | 66 |

| Fig. # | Mean | S.D. | S.E. | Statistical methods used | P value | # samples |
| --- | --- | --- | --- | --- | --- | --- |
| **Figs 6c and 6d** |  |  |  |  |  |  |
| Hourly time spent in nest boxes from 6:00 to 21:00 in weeks 40-43 (min/bird/h)-w/ one-week robot running | | | | $-$ |  |  |
| 6 | 0.81 | 0.57 | 0.07 |  | $-$ | 66 |
| 7 | 1.12 | 0.51 | 0.06 |  |  | 66 |
| 8 | 1.22 | 0.55 | 0.07 |  |  | 66 |
| 9 | 1.07 | 0.53 | 0.06 |  |  | 66 |
| 10 | 1.02 | 0.47 | 0.06 |  |  | 66 |
| 11 | 0.92 | 0.59 | 0.07 |  |  | 66 |
| 12 | 0.76 | 0.62 | 0.08 |  |  | 66 |
| 13 | 0.59 | 0.45 | 0.06 |  |  | 66 |
| 14 | 0.71 | 0.54 | 0.07 |  |  | 66 |
| 15 | 0.60 | 0.51 | 0.06 |  |  | 66 |
| 16 | 0.54 | 0.52 | 0.06 |  |  | 66 |
| 17 | 0.40 | 0.36 | 0.04 |  |  | 66 |
| 18 | 0.35 | 0.34 | 0.04 |  |  | 66 |
| 19 | 0.34 | 0.36 | 0.04 |  |  | 66 |
| 20 | 0.65 | 0.90 | 0.11 |  |  | 66 |
| 21 | 8.89 | 10.41 | 1.28 |  |  | 66 |
| Hourly time spent in nest boxes from 6:00 to 21:00 in weeks 40-43 (min/bird/h)-w/ two-week robot running | | | | $-$ |  |  |
| 6 | 0.88 | 0.59 | 0.07 |  | $-$ | 66 |
| 7 | 1.02 | 0.49 | 0.06 |  |  | 66 |
| 8 | 1.04 | 0.49 | 0.06 |  |  | 66 |
| 9 | 1.11 | 0.64 | 0.08 |  |  | 66 |
| 10 | 1.09 | 0.72 | 0.09 |  |  | 66 |
| 11 | 0.88 | 0.57 | 0.07 |  |  | 66 |
| 12 | 0.73 | 0.63 | 0.08 |  |  | 66 |
| 13 | 0.78 | 0.57 | 0.07 |  |  | 66 |
| 14 | 0.80 | 0.67 | 0.08 |  |  | 66 |
| 15 | 0.65 | 0.49 | 0.06 |  |  | 66 |
| 16 | 0.58 | 0.57 | 0.07 |  |  | 66 |
| 17 | 0.60 | 0.66 | 0.08 |  |  | 66 |
| 18 | 0.55 | 0.47 | 0.06 |  |  | 66 |
| 19 | 0.51 | 0.49 | 0.06 |  |  | 66 |
| 20 | 0.86 | 0.61 | 0.08 |  |  | 66 |
| 21 | 10.29 | 11.27 | 1.39 |  |  | 66 |

**Minimal Data Set-Tables**

| Table. # | Mean | S.D. | S.E. | Statistical methods used | P value | # samples |
| --- | --- | --- | --- | --- | --- | --- |
| **Table 1** |  |  |  |  |  |  |
| Weekly floor egg rate (%) | | | | Two-way ANOVA using PROC MIXED statement, LSD comparison | 0.57  <0.01 |  |
| w/o robot running | 32.6 | 23.3 | 3.7 |  |  | 40 |
| w/ one-week robot running | 46.5 | 20.4 | 3.2 |  |  | 40 |
| w/ two-week robot running | 40.9 | 21.6 | 3.4 |  |  | 40 |
| 34 | 60.9 | 20.8 | 6.0 |  |  | 12 |
| 35 | 49.1 | 23.5 | 6.8 |  |  | 12 |
| 36 | 39.6 | 20.1 | 5.8 |  |  | 12 |
| 37 | 38.8 | 23.7 | 6.8 |  |  | 12 |
| 38 | 36.6 | 24.2 | 7.0 |  |  | 12 |
| 39 | 42.0 | 25.2 | 7.3 |  |  | 12 |
| 40 | 32.9 | 18.2 | 5.2 |  |  | 12 |
| 41 | 33.3 | 19.9 | 5.8 |  |  | 12 |
| 42 | 33.9 | 19.6 | 5.6 |  |  | 12 |
| 43 | 32.9 | 19.1 | 5.5 |  |  | 12 |
| Relative floor egg reduction (%) | | | |  | 0.90  0.12 |  |
| w/o robot running | 23.7 | 31.9 | 5.6 |  |  | 32 |
| w/ one-week robot running | 24.1 | 13.2 | 2.3 |  |  | 32 |
| w/ two-week robot running | 31.1 | 27.5 | 4.9 |  |  | 32 |
| 34 | $-$ | $-$ | $-$ |  |  | $-$ |
| 35 | 22.3 | 18.0 | 5.2 |  |  | 12 |
| 36 | 36.5 | 20.9 | 6.0 |  |  | 12 |
| 37 | 40.7 | 24.7 | 7.1 |  |  | 12 |
| 38 | 44.2 | 27.7 | 8.0 |  |  | 12 |
| 39 | $-$ | $-$ | $-$ |  |  | $-$ |
| 40 | 16.3 | 17.6 | 5.1 |  |  | 12 |
| 41 | 18.9 | 23.2 | 6.7 |  |  | 12 |
| 42 | 14.9 | 25.0 | 7.2 |  |  | 12 |
| 43 | 16.9 | 29.2 | 8.4 |  |  | 12 |
| **Table 2** |  |  |  |  |  |  |
| Hen-day egg production (%) | | | | Two-way ANOVA using PROC MIXED statement, LSD comparison | 0.99 |  |
| w/o robot running | 86.5 | 13.2 | 2.1 |  |  | 40 |
| w/ one-week robot running | 85.7 | 10.4 | 1.6 |  |  | 40 |
| w/ two-week robot running | 85.6 | 8.7 | 1.4 |  |  | 40 |

| Table. # | Mean | S.D. | S.E. | Statistical methods used | P value | # samples |
| --- | --- | --- | --- | --- | --- | --- |
| **Table 2** |  |  |  |  |  |  |
| Hen-day egg production (%) | | | | Two-way ANOVA using PROC MIXED statement, LSD comparison | <0.01 | 12 |
| 34 | 77.6 | 11.6 | 3.3 |  |  | 12 |
| 35 | 86.9 | 4.7 | 1.3 |  |  | 12 |
| 36 | 86.0 | 7.0 | 2.0 |  |  | 12 |
| 37 | 80.6 | 4.9 | 1.4 |  |  | 12 |
| 38 | 90.0 | 15.8 | 4.6 |  |  | 12 |
| 39 | 91.7 | 20.0 | 5.8 |  |  | 12 |
| 40 | 86.5 | 7.7 | 2.2 |  |  | 12 |
| 41 | 86.3 | 8.7 | 2.5 |  |  | 12 |
| 42 | 85.4 | 8.0 | 2.3 |  |  | 12 |
| 43 | 88.3 | 5.5 | 1.6 |  |  | 12 |
| Feed intake (g/bird/day) | | | |  | 0.40  <0.01 |  |
| w/o robot running | 132.4 | 23.5 | 3.7 |  |  | 40 |
| w/ one-week robot running | 127.3 | 25.0 | 4.0 |  |  | 40 |
| w/ two-week robot running | 130.3 | 28.8 | 4.6 |  |  | 40 |
| 34 | 136.2 | 33.0 | 9.5 |  |  | 12 |
| 35 | 115.8 | 6.0 | 1.7 |  |  | 12 |
| 36 | 112.2 | 10.2 | 2.9 |  |  | 12 |
| 37 | 132.8 | 15.5 | 4.5 |  |  | 12 |
| 38 | 163.7 | 30.0 | 8.7 |  |  | 12 |
| 39 | 110.1 | 11.0 | 3.2 |  |  | 12 |
| 40 | 124.6 | 20.6 | 6.0 |  |  | 12 |
| 41 | 136.5 | 29.4 | 8.5 |  |  | 12 |
| 42 | 128.1 | 27.2 | 7.9 |  |  | 12 |
| 43 | 140.1 | 13.0 | 3.7 |  |  | 12 |
| FCR (kg feed/dozen eggs) | | | |  | 0.92  <0.01 |  |
| w/o robot running | 1.75 | 0.27 | 0.04 |  |  | 40 |
| w/ one-week robot running | 1.73 | 0.25 | 0.04 |  |  | 40 |
| w/ two-week robot running | 1.78 | 0.24 | 0.04 |  |  | 40 |
| 34 | 1.76 | 0.15 | 0.04 |  |  | 12 |
| 35 | 1.43 | 0.07 | 0.02 |  |  | 12 |
| 36 | 1.46 | 0.14 | 0.04 |  |  | 12 |
| 37 | 1.91 | 0.09 | 0.03 |  |  | 12 |
| 38 | 1.86 | 0.34 | 0.10 |  |  | 12 |
| 39 | 1.79 | 0.13 | 0.04 |  |  | 12 |
| 40 | 1.81 | 0.16 | 0.05 |  |  | 12 |
| 41 | 1.87 | 0.21 | 0.06 |  |  | 12 |
| 42 | 1.90 | 0.28 | 0.08 |  |  | 12 |
| 43 | 1.76 | 0.15 | 0.04 |  |  | 12 |

| Table. # | Mean | S.D. | S.E. | Statistical methods used | P value | # samples |
| --- | --- | --- | --- | --- | --- | --- |
| **Table 3** |  |  |  |  |  |  |
| Nest egg mass (g/egg) | | | | One-way ANOVA using PROC MIXED statement, LSD comparison | 0.05 |  |
| w/o robot running | 62.9 | 1.1 | 0.4 |  |  | 8 |
| w/ one-week robot running | 63.5 | 2.0 | 0.7 |  |  | 8 |
| w/ two-week robot running | 61.7 | 0.4 | 0.1 |  |  | 8 |
| Floor egg mass (g/egg) | | | |  | 0.13 |  |
| w/o robot running | 60.2 | 2.9 | 1.0 |  |  | 8 |
| w/ one-week robot running | 62.2 | 2.0 | 0.7 |  |  | 8 |
| w/ two-week robot running | 62.2 | 1.5 | 0.5 |  |  | 8 |
| Overall (g/egg) | | | |  | 0.04 |  |
| w/o robot running | 62.6 | 0.9 | 0.3 |  |  | 8 |
| w/ one-week robot running | 63.2 | 1.8 | 0.6 |  |  | 8 |
| w/ two-week robot running | 61.7 | 0.4 | 0.1 |  |  | 8 |
| **Table 4** |  |  |  |  |  |  |
| Foot pad score | | | | $-$ | $-$ |  |
| w/o robot running | 0 | 0 | 0 |  |  | 4 |
| w/ one-week robot running | 0 | 0 | 0 |  |  | 4 |
| w/ two-week robot running | 0 | 0 | 0 |  |  | 4 |
| Mortality (%) | | | | One-way ANOVA using PROC MIXED statement, LSD comparison | 0.32 |  |
| w/o robot running | 0.8 | 1.7 | 0.8 |  |  | 4 |
| w/ one-week robot running | 0 | 0 | 0 |  |  | 4 |
| w/ two-week robot running | 1.7 | 1.9 | 1.0 |  |  | 4 |
| **Table 5** |  |  |  |  |  |  |
| Mean (pg/ml) | | | | One-way ANOVA using PROC MIXED statement, LSD comparison | 0.21  0.07 |  |
| w/o robot running | 236.9 | 268.6 | 95.0 |  |  | 8 |
| w/ one-week robot running | 265.0 | 302.7 | 107.0 |  |  | 8 |
| w/ two-week robot running | 215.9 | 263.3 | 93.1 |  |  | 8 |
| 34 | 147.2 | 44.5 | 12.8 |  |  | 12 |
| 38 | 382.7 | 399.2 | 115.2 |  |  | 12 |
| CV (%) | | | |  | 0.07  0.17 |  |
| w/o robot running | 63.2 | 33.9 | 12.0 |  |  | 8 |
| w/ one-week robot running | 65.7 | 33.9 | 12.0 |  |  | 8 |
| w/ two-week robot running | 64.3 | 35.4 | 12.5 |  |  | 8 |
| 34 | 77.2 | 32.2 | 9.3 |  |  | 12 |
| 38 | 54.3 | 32.7 | 9.4 |  |  | 12 |
| **Table 6** |  |  |  |  |  |  |
| Bone breaking force (kg) | | | | Two-way ANOVA using PROC MIXED statement, LSD comparison | 0.89  0.22 |  |
| w/o robot running | 24.8 | 2.2 | 0.8 |  |  | 8 |
| w/ one-week robot running | 24.8 | 2.1 | 0.7 |  |  | 8 |
| w/ two-week robot running | 24.9 | 2.3 | 0.8 |  |  | 8 |
| 34 | 24.2 | 2.7 | 0.8 |  |  | 12 |
| 42 | 25.3 | 1.1 | 0.3 |  |  | 12 |

| Table. # | Mean | S.D. | S.E. | Statistical methods used | P value | # samples |
| --- | --- | --- | --- | --- | --- | --- |
| **Table 6** |  |  |  |  |  |  |
| Fresh bone weight (g) | | | | Two-way ANOVA using PROC MIXED statement, LSD comparison | 0.20  0.39 |  |
| w/o robot running | 11.0 | 0.5 | 0.2 |  |  | 8 |
| w/ one-week robot running | 11.0 | 0.6 | 0.2 |  |  | 8 |
| w/ two-week robot running | 11.0 | 0.5 | 0.2 |  |  | 8 |
| 34 | 10.9 | 0.7 | 0.2 |  |  | 12 |
| 42 | 11.1 | 0.4 | 0.1 |  |  | 12 |
| Dried bone weight (g) | | | |  |  |  |
| w/o robot running | 7.2 | 0.3 | 0.1 |  | 0.27  0.44 | 8 |
| w/ one-week robot running | 7.3 | 0.3 | 0.1 |  |  | 8 |
| w/ two-week robot running | 7.2 | 0.3 | 0.1 |  |  | 8 |
| 34 | 7.2 | 0.4 | 0.1 |  |  | 12 |
| 42 | 7.3 | 0.1 | 0.1 |  |  | 12 |
| Bone ash weight (g) | | | |  |  |  |
| w/o robot running | 4.0 | 0.3 | 0.1 |  | 0.64  0.83 | 8 |
| w/ one-week robot running | 4.1 | 0.3 | 0.1 |  |  | 8 |
| w/ two-week robot running | 4.0 | 0.2 | 0.1 |  |  | 8 |
| 34 | 4.0 | 0.4 | 0.1 |  |  | 12 |
| 42 | 4.1 | 0.1 | 0.1 |  |  | 12 |
| Ash percentage (%) | | | |  |  |  |
| w/o robot running | 55.9 | 2.1 | 0.7 |  | 0.98  0.67 | 8 |
| w/ one-week robot running | 56.0 | 2.1 | 0.7 |  |  | 8 |
| w/ two-week robot running | 55.6 | 1.8 | 0.6 |  |  | 8 |
| 34 | 56.2 | 3.0 | 0.9 |  |  | 12 |
| 42 | 55.7 | 0.2 | 0.1 |  |  | 12 |
| **Table 7** |  |  |  |  |  |  |
| Bone breaking force (%) | | | | One-way ANOVA using PROC MIXED statement, LSD comparison |  |  |
| w/o robot running | 26.1 | 3.8 | 1.9 |  | 0.31 | 4 |
| w/ one-week robot running | 26.4 | 3.5 | 1.8 |  |  | 4 |
| w/ two-week robot running | 26.2 | 4.3 | 2.1 |  |  | 4 |
| Fresh bone weight (%) | | | |  |  |  |
| w/o robot running | 9.4 | 1.0 | 0.5 |  | 0.88 | 4 |
| w/ one-week robot running | 9.5 | 1.1 | 0.6 |  |  | 4 |
| w/ two-week robot running | 9.4 | 1.0 | 0.5 |  |  | 4 |
| Dried bone weight (%) | | | |  |  |  |
| w/o robot running | 11.3 | 0.9 | 0.4 |  | 0.77 | 4 |
| w/ one-week robot running | 11.3 | 0.8 | 0.4 |  |  | 4 |
| w/ two-week robot running | 11.2 | 0.9 | 0.5 |  |  | 4 |
| Bone ash weight (%) | | | |  |  |  |
| w/o robot running | 13.5 | 0.8 | 0.4 |  | 0.97 | 4 |
| w/ one-week robot running | 13.5 | 0.8 | 0.4 |  |  | 4 |
| w/ two-week robot running | 13.4 | 0.9 | 0.4 |  |  | 4 |

| Table. # | Mean | S.D. | S.E. | Statistical methods used | P value | # samples |
| --- | --- | --- | --- | --- | --- | --- |
| **Table 7** |  |  |  |  |  |  |
| Ash percentage (%) | | | | One-way ANOVA using PROC MIXED statement, LSD comparison |  |  |
| w/o robot running | 5.4 | 0.7 | 0.3 |  | 0.98 | 4 |
| w/ one-week robot running | 5.5 | 0.7 | 0.3 |  |  | 4 |
| w/ two-week robot running | 5.4 | 0.7 | 0.3 |  |  | 4 |
| **Table 8** |  |  |  |  |  |  |
| Time spent in nest boxes from 7:00 to 13:00 (min/bird/day) | | | | Two-way ANOVA using PROC MIXED statement, LSD comparison |  |  |
| w/o robot running | 6.6 | 2.0 | 0.3 |  | 0.77  <0.01 | 40 |
| w/ one-week robot running | 6.2 | 1.7 | 0.3 |  |  | 40 |
| w/ two-week robot running | 6.9 | 2.3 | 0.4 |  |  | 40 |
| 34 | 7.4 | 1.8 | 0.5 |  |  | 12 |
| 35 | 6.8 | 2.0 | 0.6 |  |  | 12 |
| 36 | 8.6 | 3.7 | 1.1 |  |  | 12 |
| 37 | 7.0 | 1.7 | 0.5 |  |  | 12 |
| 38 | 6.1 | 1.5 | 0.4 |  |  | 12 |
| 39 | 6.1 | 1.8 | 0.5 |  |  | 12 |
| 40 | 6.3 | 1.3 | 0.4 |  |  | 12 |
| 41 | 6.0 | 1.6 | 0.5 |  |  | 12 |
| 42 | 5.7 | 0.9 | 0.3 |  |  | 12 |
| 43 | 5.8 | 1.4 | 0.4 |  |  | 12 |
| Time spent in nest boxes during a day (min/bird/day) | | | |  |  |  |
| w/o robot running | 12.9 | 5.5 | 0.9 |  | 0.52  <0.01 | 40 |
| w/ one-week robot running | 13.0 | 5.0 | 0.8 |  |  | 40 |
| w/ two-week robot running | 16.1 | 6.4 | 1.0 |  |  | 40 |
| 34 | 14.6 | 4.1 | 1.2 |  |  | 12 |
| 35 | 14.9 | 6.8 | 2.0 |  |  | 12 |
| 36 | 19.2 | 11.6 | 3.4 |  |  | 12 |
| 37 | 15.4 | 5.3 | 1.5 |  |  | 12 |
| 38 | 14.4 | 4.2 | 1.2 |  |  | 12 |
| 39 | 13.0 | 3.8 | 1.1 |  |  | 12 |
| 40 | 13.2 | 4.0 | 1.2 |  |  | 12 |
| 41 | 12.5 | 3.7 | 1.1 |  |  | 12 |
| 42 | 11.3 | 2.9 | 0.8 |  |  | 12 |
| 43 | 11.4 | 3.5 | 1.0 |  |  | 12 |
