# Supplementary material for: Effects of ground robot manipulation on hen floor egg reduction, production performance, stress response, bone quality, and behavior
Source: PLoS One. 2022 Apr 22;17(4):e0267568. doi: 10.1371/journal.pone.0267568 (PMC9032375; doi:10.1371/journal.pone.0267568)
Supplement: Attachment — Submitted filename: Response to Reviewers.docx [file pone.0267568.s003.docx]

Dear editor and reviewers,

On behalf of the co-authors, we are very grateful for the valuable comments from you and for giving us the

opportunity to further improve our manuscript. To be honest, we were overwhelmed by the 114 comments in the previous review and focused most of our attention on addressing the comments technically. We also realized our language may not meet the requirement of publications; therefore, we improved the relevant contents proposed by the reviewers and invited the native English speakers within the research group to proofread the paper. The revised portions can be found in the track-change manuscript, and the line numbers in this response file are referred to in the clean-version manuscript.

Special thanks to the Reviewers for their valuable remarks and comments again. We also earnestly appreciate the Editor’s diligent work. We hope that the corrections will meet your approval and our paper

will be finally accepted for publication in *PLOS ONE*. Looking forward to hearing from you!

Best Regards,

Guoming Li.

**Redundancy and readability**.

**Additional Editor Comments**

Authors should pay more attention to revise based the reviewer's comments.

Response 1: We have carefully gone through the reviewers’ comments again and made sure that everything is correct and accurate.

Highlight the revised sections.

Response 2: The previous revisions were highlighted in yellow text, while the current revisions were highlighted in blue font.

Suggested to revise the manuscript once again for grammatical errors and to improve language efficiency. The manuscript should be proofread by native speaker for the correction of English language so as to meet the standard of publication in the journal.

Response 3: Grammatical errors and language efficiency were double-checked and proofread by the native English speaker within the research group.

**Reviewer #1:**

The quality of the scientific writing is not suitable for publication and I encourage the (presumably) native English co-authors to review the added text.

Response 4: We have invited the native English co-authors to review the added text.

Lines 41-43: The first part of the sentence you make a general statement, the second part is specific to the experiment, please check and revise.

Response 5: The second part was removed to avoid any confusion.

~~however, such an effect diminished as hens became familiarized with the robot~~

Line 54: on the littered floor.

Response 6: It was changed to ‘…on the littered floor’ (L48)

Line 78: may have potential for reducing

Response 7: Relevant contents were removed to improve language accuracy.

~~and robotics may have potential for reducing floor eggs of hens in CF housing systems but should be evaluated~~

Line 83: on commercial farms.

Response 8: It was changed to ‘…on commercial farms’ (L76).

Lines 83-84: I don’t think you have to state what the benefits of a controlled experiment are. Most researchers will know the differences between a controlled trial and one on a commercial farm.

Response 9: The statement of describing benefits of floor pens was removed.

~~Therefore, floor pens were set for the preliminary examinations because of controlled environments, convenient management with limited labor, and so on.~~

I’m not sure stating the measures were borrowed from broilers is correct. You can still measure these in laying hens, they are part of laying hen welfare assessments. My previous issue was why only these measures when you are testing laying hens not broilers. Also this new section needs to be checked to make sure the grammar is correct.

Response 10: Thanks for pointing this out. We rephrased the contents as follows. And grammar was double-checked to ensure language accuracy.

“Robot manipulation may encourage hen walking behaviors and enhance litter aeration via me-chanical disturbance of litter shavings during turning operations as a result of skid steer locomotion and the associated traction force dynamics experienced while turning. Litter moisture may be re-duced due to decreased hen lying time and improved ventilation in the aerated litter. While not commonly measured in a short-term trial for laying hens, litter moisture content differed signifi-cantly from various treatments for 34-week laying hens in an eight-week experiment [17]. Hen footpad health may benefit from the potentially improved litter conditions and can be reflected by one of the representative footpad measures [18] like footpad score determined by footpad derma-titis rates [19]. Although young hens are not likely to have footpad dermatitis, Campbell et al. [20] reported a 0.3% footpad dermatitis rate for free-range hens at 20-36 weeks of age.” (L90-L100)

Lines 80-105. I’m not sure about the layout of this section. It is strange to see such a lengthy description of essentially your methods. Could this section be split up and incorporated into the above text to have a description of previous findings followed by what is missing. Then you can finalise the section with a statement of your objectives. Also, the writing could benefit from a proper check by the co-authors who I presume are native English speakers. The writing is not as good as it should be for publication.

Response 11: We have revised the section according to this comment. The section was split up into one paragraph describing the necessity of the measures and another paragraph to state the objective of this study. Grammar was checked and details can be found in L75-L105.

Line 112: one commercial farm or multiple commercial farms.

Response 12: The birds were from two commercial farms. (L111)

Could you include some explanation here as to why you used birds of 34 weeks and not young pullets. While I appreciate your response to my previous comment, other readers need to see some explanation of why such old birds were used.

Response 13: Some descriptions were added to indicate the reason of selecting 34-week-old birds rather than young pullets.

“Older birds (34 weeks of age) rather than young pullets were recommended by the industry farm managers, because they may be more likely to overcome the challenges of transportation stress, cold stress (at the end of October), and adaptation to new housing environments.” (L113-L116)

Line 114: from a commercial farm

Response 14: It was changed to “..from two commercial farms”. (L111)

Line 120: enriched not enrich

Response 15: It was changed to ‘enriched’. (L121)

Line 126-130: Same comment as previously, the new text needs to be read by the native speakers as the grammar is not correct. Likely this comment applies throughout the manuscript to all new text that has been added.

Response 16: All added texts were proofread by the native speakers. Changes can be found in the track-change manuscript.

Line 142: each not wach. I recommend a more detailed check of your manuscript before resubmitting it again.

Response 17: It was changed to ‘each’. (L141)

Line 151: ‘explained later’ reads colloquially. Please refer to a specific section and reword.

Response 18: It was changed to ‘Section 2.3.4 Nesting behavior monitoring’ (L149-L150)

Line 165: ‘footpad’ rather than just ‘foot’ if you observed the footpad only.

Response 19: It was changed to ‘footpad’ throughout the manuscript.

Line 167: why only in flock 2?

Response 20: The data collection was conducted in the two flocks. However, due to unexpected events, egg mass data in flock 1 was lost, and only flock 2 data were used for further analysis. (L163-164)

Line 168: were weighed weekly.

Response 21: It was changed to ‘…were weighed weekly’ (L165).

Lines 177-183: an explanation for including this measurement is better placed within the introduction, although not with this exact wording in the intro.

Response 22: The explanation for including the litter moisture content measurement was placed within the introduction and rephrased. Details can be found in L75-L100.

Line 184: I’m not completely convinced by your explanation of ‘too many measurements’. If you are assessing a bird for their footpads, you can easily do a complete WQ assessment on that same bird in approximately 1 min (I’ve done plenty myself). I’ve run many laying hen trials that encompass many measurements. Perhaps the wording could be changed here to state you focussed on measures you predicted to be most affected by your treatment. Please avoid colloquial text, please get all additional text checked by a native speaker as the scientific writing is currently not of a standard for publication.

Response 23: We may mislead the reviewer about stating ‘too many measurements’. Yes, measuring footpad of one hen was really quick, however, besides that, we need to euthanize birds, take bone samples, obtain the weights of feed, eggs, and birds, and count eggs at the end of this experiment. The farm manager only allowed us to visit the farm and complete these tasks within the same day because of biosecurity issue. That means a lot for us. Therefore, we selected one of the representative measures, footpad score, to reflect footpad health while avoiding overloading.

Similar to litter moisture content, we also moved the description about rationale of selecting the footpad score measure to introduction and rephrased the contents. Colloquial texts were rephrased to meet the standard of publication. Detailed rationale of the two measures can be found in the introduction section. can be found in L90-L100.

Lines 195-196: you need a clearer explanation of what was being observed in terms of ‘bird health status’ and why. Clarify the observers were assessing potential stress levels of the birds.

Response 24: The poultry scientist in our research group helped us to clarify that.

“All birds for blood sampling were handled similarly to avoid introducing additional stress during collection. The physiological parameters (i.e., heart rate, respiratory rate, body temperature, and mucous membrane color) were observed and detected by the poultry scientists before blood sam-pling. This approach ensured that all assessed birds were healthy and minimized confounding factors or pathological conditions that would otherwise affect the results.” (L177-L182)

Lines 218-221: please check grammar, the wording seems strange to me. Unsure if there is a part missing in this sentence. ‘and then the remaining in a crucible’ ?

Response 25: The sentences were revised to the following (L201-203).

“The oven-dried bone was weighed, placed in a crucible, and oven-dried again in a furnace (Isotemp D3714 muffle furnace, Thermo Fisher Scientific Inc., Waltham, MA) for 24 hours at 600 °C. After the last drying, the remaining content was weighed to obtain ash weights of bones.”

Line 320: how was it validated? Do you have data on that? Are you sure there was no detection range for your passive tag? Are you claiming the passive tags could be read at any distance? If they were validated to only be recording hens in the nest box then I presume there was actually a detection range for them.

Response 26: After the hardware was installed, an RFID tag was held near the antenna, and d the interface of a free visualization software, MultiReader for SpeedWay Gen2 RFID (Version 6.6.11.240), was ob-served. Once the RFID tag was within the detection range of the antenna, readings were displayed on the interface and the system power was adjusted, so that the system was validated to only record birds located inside a nest box rather than birds standing next to a nest box. The free software was only for real-time data visualization and could not store validation data. It should be noted that passive tags neither have an internal power source nor actively emit signals. They utilize electro-magnetic waves received from a reader. Once the reader transmits signals to a tag, the connected antenna creates a magnetic field and the tag circuit uses the power generated to transmit data back to the reader. Theoretically, a passive tag does not have a detection range, but the connected antenna had one within 80 cm based on current RFID settings and power supply (L211-L221). Therefore, we just need to validate the power of the system to ensure the antenna atop a nest box for only covering hens in the box.

Line 230: please state ‘attached’ rather than ‘bonded’. Bonded implies the tag was stuck to the leg, not attached to the leg.

Response 27: Thanks. It was changed to ‘attached’. (L210)

Line 260: were used to reflect bird stress responses to the robot

Response 28: It was changed to ‘…were used to reflect bird stress responses to the robot’. (L249).

Lines 291-195: Are these total sample sizes you are listing or per pen? Please make sure it is clear for the reader.

Response 29: It was changed to ‘The sample size (n) for each treatment per pen was…’ (L273).

Line 297: So then did you remove non-significant interactions from your final models?

Response 30: We did not remove non-significant interactions from the final models but just not report the interaction results. (L282)

Line 325: footpad

Response 31: It was changed to ‘footpad’ throughout the manuscript.

Line 473: is nest use really a side effect in this trial? Also, the use of ‘side effect’ is colloquial.

Response 32: The ‘side effect’ was removed from the article. The sentence was changed to “This research was mainly aimed to investigate the effects of ground robot manipulation on hen floor egg reduction, production performance, stress response, bone quality, and nesting behaviors.” (L444-445)

Discussion: I did not intend for you to take my previous comments word for word and add them into your manuscript. Please check through the discussion for colloquial wording to improve the scientific writing quality.

Response 33: We understand that the reviewer does not intend to ask us for including the comment words, but we thought these wordings can strengthen the scientific values of the discussion. Therefore, we would kindly request these wordings to be included into the discussion. But if this causes any ethical problem, we will remove the relevant contents. The whole discussion section was rephrased to enhance the article quality.

**Reviewer #2:**

First of all I would like to point out that the authors did a great job to try to answer and include the comments of the reviewers in the revised version of the paper. However, this not resulted in a better version of the paper which I will try to explain below. I am afraid the current version has not the quality needed of a version which can be published. I would advise the authors to let the paper be screened by experienced writers (senior researchers) both for language and scientific content (esp. the discussion section, which is long, and not to the point at many places). I give some examples below.

Response 34: We appreciated the reviewer’s compliment on our previous revision and realized that we did not do well in the language. The senior researchers in the research group have proofread this article and made sure everything is accurate and concise.

Line 30: adding that it now has 4 replicates still does not clarify the setup. I would like to see here a better explanation: each flock has 6 pens with 3 treatments, resulting in 2 replicates per treatment per flock. As a total of 2 flocks was studied, each treatment had a total of 4 replicates. Please adjust the text.

Response 35: Thanks. The relevant contents were rephrased according to the reviewer’s comment.

“Two successive flocks of 180 Hy-Line Brown hens at 34 weeks of this age were used. The treatment structure for each flock consisted of six pens with three treatments (without robot running, with one-week robot running, and with two-weeks robot running), resulting in two replicates per treatment per flock and four replicates per treatment with two flocks.” (L26-L30)

Line 12, same here. Please indicate that this were two successive flocks, and that in each flock 6 pens were present for four treatments. so then it is clear how you end up with 4 replicates.

Response 36: The ‘two successive flocks’ and ‘six pens in each flock’ are added and indicated in L27-29.

Line 28 ‘human’ should be ‘humans’ . I have the feeling that the text has not been checked by a native speaker and I would encourage the authors to do so. There are more textual errors, I might have missed errors but please check the text.

Response 37: It was changed to ‘technicians’ (L128).

Line 32, typo, chores should be scores

Response 38: To avoid confusion, the ‘chores’ was changed to ‘tasks’ (L131)

Line 42 same, wach should be each

Response 39: ‘wach’ was changed to ‘each’. (L141)

Line 75 mutually verified. It is not clear to me what you mean herel.

Response 40: To avoid confusion, the sentence was changed ‘…evaluation results were verified between observers’ (L171-L172)

Lie 84 I am happy with a detailed answer to the question, but this argument should not have a place here but in the introduction and the text should be shortened. Further I am not convinced by this answer and simply stating that you had too many measures and should select one is not a good argument. You can better argue why you think this specific parameter is affected by the robot.

Response 41: We rephrased the rationale statements of selecting footpad scores and litter moisture content and place them in the introduction. (L90-L100)

Robot manipulation may encourage hen walking behaviors and enhance litter aeration via me-chanical disturbance of litter shavings during turning operations as a result of skid steer locomotion and the associated traction force dynamics experienced while turning. Litter moisture may be re-duced due to decreased hen lying time and improved ventilation in the aerated litter. While not commonly measured in a short-term trial for laying hens, litter moisture content differed signifi-cantly from various treatments for 34-week laying hens in an eight-week experiment [17]. Hen footpad health may benefit from the potentially improved litter conditions and can be reflected by one of the representative footpad measures [18] like footpad score determined by footpad derma-titis rates [19]. Although young hens are not likely to have footpad dermatitis, Campbell et al. [20] reported a 0.3% footpad dermatitis rate for free-range hens at 20-36 weeks of age.

Line 262 replace to build by ‘Week 34 corticosterone concentrations were considered as baseline concentrations after adaptation to the new housing’

Response 42: The sentence was changed to “Week 34 corticosterone concentrations were considered baselines after birds adapted to the new housing”. (L174-L175)

Statistical paragraph: sample size can be included in the results. This should be described better, you can simply say that log transformation was used to approach normal distribution.

Response 43: The sample size were indeed included in each table notes. And the other reviewer required us to summarize the sample size, and we may kindly request to maintain these sample size summary in the statistical paragraph.

The relevant contents were revised to “… approach normal distribution”. (L272)

Line 478 remove ‘and’ at the beginning of the sentence.

Response 44: ‘And’ was removed. (L450).

~~And~~ placing them into new…

Line 482 should be floor egg percentage and indicate should be indicates

Response 45: The sentence was changed to ‘…high floor egg rates…’ (L451).

Line 301 and further, this should not be in the discussion. I understand your experiment was planned, but this argumentation is not convincing.

Response 46: We understand the reviewer’s concern about more robot manipulation resulting in less floor eggs. However, like we debate previously that ‘The whole experiment including the robot running procedure were planned and scheduled before it was started. We need to strictly execute the experimental plan to obtain statistical results, even though we guessed that more robot running may reduce floor eggs more’. Although this argumentation is not convincing, the only way we can do is to call for further experiment to verify this hypothesis. To avoid colloquial texts, we also removed relevant contents.

Line 315. I think there are behaviors that can indicate whether or not hens are used to the robot. Distance to robot is one, also home pen behaviour (activity, restlessness) and fear responses can be used. Please remove this text and focus on the avoidance behavior (which is an appropriate measure)

Response 47: We added some discussion, “Other behaviors, such as activity and restlessness, and fear responses may also be examined in the future for indicating bird adaptations to robots. (L477-478)

Line 405 this should not be part of the discussion. I expect here a comparison with other findings and if these are not present, a critical reflection on the result

Response 48: We are sorry that we cannot be navigated to the exact problem.

Around the Line 405 of the previous clean-version manuscript, “It should be noted that the frequency was only counted when there was at least one bird using a nest box, and the frequency for the empty nest box was not included.” are presented in the **result** section.

Around the Line 405 of the previous track-change manuscript, “Table 6 shows the means of parameters for evaluating bone quality of hens. The average bone breaking force, fresh bone weight, dried bone weight, bone ash weight, and ash percentage were 24.8 kg, 11.0 g, 7.3 g, 4.1 g, and 56.0%, respectively, and they were not significantly different with the treatments (P≥0.20).” are presented in the **result** section.

Neither are these two places part of the discussion. If the reviewer can pinpoint this question, we would love to revise it accordingly.

The discussion has not been improved as compared to the first version of the paper, unfortunately. Please keep it concise, use appropriate literature if present, and try to limit it to placing your findings in the context of what already has been found. It is now difficult to read, contains information which should not be there.

Please remove the figure from the discussion. Place it in the results or in the supporting information (better option) and briefly mention the data.

Response 49: We reorganize the discussion section and proofread it. The figure indicating the bird adaptation and relevant descriptions were moved to the result sections. Although the reviewer suggested placing the figure in the supporting information be a better option, we may think the figure is informative about the adaptation process. Therefore, we kindly request to maintain this in the paper (result section). Details can be found in the result section, “Anecdotal observations for bird adaptation to the robot”.
