# Supplementary material for: Effects of ground robot manipulation on hen floor egg reduction, production performance, stress response, bone quality, and behavior
Source: PLoS One. 2022 Apr 22;17(4):e0267568. doi: 10.1371/journal.pone.0267568 (PMC9032375; doi:10.1371/journal.pone.0267568)
Supplement: Attachment — Submitted filename: Response to Reviewers.docx [file pone.0267568.s004.docx]

Dear editor and reviewers,

On behalf of the co-authors, I would like to thank you for your hard work again, especially for reviewer #1. I realized how much time the reviewer input for reviewing this manuscript and appreciated his/her insightful comments helping us improve language accuracy in this round. I enjoyed working on these comments and interacting with the reviewer and learned a lot from them. The changes are highlighted in the manuscript, and the response are in blue font in this file.

Best Regards,

Guoming Li.

**Reviewer #1:**

Line 51: If not rapidly collected OR If not collected in a timely manner

Response 1: It was changed to ‘If not collected in a timely manner’ (L51).

Line 63: I presume this should be ‘has not yet’

Response 2: It was changed to ‘has not yet’ (L63).

Line 64: Researchers have investigated (this would read better)

Response 3: It was changed to ‘Researchers have investigated’ (L64).

Line 77: preliminary trials were conducted in experimental pens

Response 4: It was changed to ‘preliminary trials were conducted in experimental pens’ (L77).

Line 78: ‘performance of floor eggs’ is strange wording. Could it be adjusted to be ‘performance of floor egg laying’ or ‘interested in floor egg laying and other production indicators’

Response 5: It was changed to ‘performance of floor egg laying’ (L78-79).

Lines 81-85: please reword this sentence, it is long and awkwardly worded

Response 6: The sentence was revised as follows.

‘Yang et al. [10] elaborated that robot operations encouraged broiler movement, and increasing bird activities have been correlated to improved bird bone quality in loose housing systems [3, 14]. Therefore, the robot was assumed to increase hen activities in this study and consequently enhance bird bone quality.’ (L81-85).

Lines 75-100: I think this whole section could be streamlined. It is currently very wordy and the points could be made more succinctly. Litter effects may make more sense placed after the bone quality statement as both mention hen movement.

Response 7: We reorganized this paragraph and removed the redundancy of description. The paragraph is inevitably lengthy because we want to justify every measure is reasonable. The litter moisture contents were placed after the bone quality statement per suggestions. The reorganized paragraph can be found in L75-96.

Line 85: It is not clear whether you mean the robot operation itself is a visual/audio alert, or if you are stating this is something that the robot does – i.e., it emits specific sounds to trigger the hens to use the laying boxes?

Response 8: It was changed to ‘Ground robots can emit sounds and lights having visual and/or audible alert capabilities and encouraging birds to utilize nest boxes’ (L91-92).

Line 102: specifically nesting behavior only? You mention hen activity in the previous section, but you didn’t directly measure that behavior (from memory?), so clarify it is just nesting behavior here in the objectives.

Response 9: It was changed to ‘and nesting behavior’ (L98).

Line 127: ‘adverse’ would be a better word than ‘inappropriate’

Response 10: It was changed to ‘adverse’ (L122).

Line 195: At the end of the experiments,

Response 11: It was changed to ‘At the end of the experiments’ (L190).

Line 196: ‘after euthanasia’ is not necessary, you already state they were euthanized,

Response 12: The words were removed. ~~after euthanasia.~~ (L191).

Line 212: is there a stray ‘d’ that needs to be removed?

Response 13: The ‘d’ was removed.

Line 223: and stored in a Python-based

Response 14: It was changed to ‘and stored in a Python-based’ (218).

Lines 269-270: A constant of 1 was added to all percentage data in decimal form to eliminate negatives and then log transformed to approach normality. (you can then delete the sentence 271-272 about the log transformation

Response 15: It was changed to ‘A constant of 1 was added to all percentage data in decimal form to eliminate negatives and then log transformed to approach normality.’ Additional log transformation statement was removed. ~~The log transformation was used to make the examined data approach a normal distribution.~~ (L267-269)

Line 282: are not reported

Response 16: It was changed to ‘are not reported’ (L277).

Line 287: had no effect

Response 17: It was changed to ‘had no effect’ (L285).

Line 349: Data were

Response 18: It was changed to ‘Data were’ (L352).

Line 401: The overall trend showed the time in

Response 19: It was changed to ‘The overall trend with the three treatments showed that hourly time spent’ (L404-405).

Line 426: The overall trend with the three treatments showed hourly

Response 20: It was changed to ‘The overall trend with the three treatments showed that hourly’ (L429-430).

Line 437: At the end of the two-week robot running period,

Response 21: It was changed to ‘At the end of the two-week robot running period’ (L440).

Line 444: ‘mainly aimed’? Or ‘aimed’? You have listed all your measurements there so not sure why it is stated as ‘mainly aimed’

Response 22: That is correct, thanks. The ‘mainly’ was removed (L447).

Line 449: I would think it is ‘likely’ rather than ‘may’ have established laying patterns

Response 23: It was changed to ‘likely to have already’ (L452).

Line 527: up to two weeks after robot running.

Response 24: It was changed to ‘up to two weeks after robot running’ (L530).

Line 552: would it be better to state ‘less than a previous study using the same RFID system’? If that is what you are referring to. When you state ‘the previous study’, it implies the reader should know the specific study you are referring to.

Response 25: It was changed to ‘less than a previous study using the same RFID system’ (L555-556)
